# Coordination of hospital discharge for older people with dementia: insights from politicians and local government officials

**DOI:** 10.1186/s12877-026-07637-x

**Published:** 2026-05-19

**Authors:** Maria Andreassen, Tove Törnqvist, Johannes Österholm

**Affiliations:** Department of Health, Medicine and Caring Sciences, Linköping University, Norrköping, Sweden

**Keywords:** Care coordination, Collaboration, Dementia, Discharge process, Integrated care, Older people, Qualitative research

## Abstract

**Background:**

After hospital care, older people with dementia often require further health and social care services. Hospital discharge is a complex process in which such support is planned and coordinated. This typically requires collaboration between professionals with different responsibilities representing various authorities to ensure integrated care. In Sweden, the decentralised organisation of health and social care services may lead to variation in discharge procedures and in how collaboration across care providers and authorities is organised. Politicians and civil servants play a central role in shaping discharge practices and in organising collaboration and coordination of services within regions and municipalities. However, their perspectives remain relatively underrepresented in previous research on hospital discharges for older people with dementia.

**Methods:**

Maximum variation strategy was used to recruit four politicians and eleven local government officials. Semi-structured interviews were conducted and analysed using reflexive thematic analysis.

**Results:**

Our findings suggest that the discharge process from inpatient hospital care is governed by formal agreements outlining responsibilities between care providers. This process involves both a physical relocation and an administrative handover of responsibilities. Strategic workforce planning is essential to ensure sustained staff competence, and particular attention must be given to safeguarding the individual's representation throughout the discharge process.

**Conclusions:**

Politicians and local government officials highlight the need for clearly defined procedures and guidelines, governed by formal agreements between care providers and care authorities. The findings problematize frequent staff turnover, which undermines the development of a stable organizational culture in relation to hospital discharges. Furthermore, there is a need for experienced professionals committed to working with people with dementia, applying a person-centred approach throughout discharges.

## Background

Discharge from hospital inpatient care is a complex process that needs to consider the patients’ medical situation, availability of services, and communication between different care providers [1–6]. Dementia is an umbrella term for cognitive and behavioral symptoms associated with various progressive brain diseases that affect higher cognitive functions and predominantly affects older people [7]. As dementia progresses to its later stages, individuals often develop complex health and social care needs [8]. These needs encompass medical, social, and behavioral challenges that require both costly and long-term health and social care services [8, 9]. These complex needs arise not only from dementia itself but also from co-existing conditions, or comorbidities [10, 11], causing delayed hospital discharges [12]. This complexity presents significant challenges for coordination and collaboration among multiple care-providing authorities and agencies involved in addressing the health and care needs of older people with cognitive impairments [3]. This, in turn, places pressure on the health and social care system to provide adequate services [13], as well as on those involved in governing these systems.

Fragmentation of health and social care services, as a consequence of divided responsibilities between different authorities, is a commonly discussed issue [5, 14–16], accentuated during the COVID-19 pandemic [16, 17]. Fragmented care pathways and limited collaboration between different care agencies contribute to discharge delays, increased risks during transitions, and greater reliance on informal caregivers, intensifying existing gaps in service delivery [5]. A commonly discussed solution to address fragmentation in the organization of healthcare and social care is collaboration between different care agencies and coordination of services [18, 19]. Collaboration refers to joint efforts that transcend organizational, professional, and sectoral boundaries and involve multiple agencies. Furthermore, coordination represents a proactive approach that brings care professionals and providers together to ensure that individuals receive integrated care across various care settings [20]. Several barriers to interprofessional collaboration and coordination of health and care services for older people with dementia have been identified in previous research [3–5]. These barriers affect hospital discharge processes and include, for example, unclear professional roles and poorly defined responsibilities [3]. This lack of clarity can lead to limited personal accountability amongst professionals [2]. Even though interest in research about coordination of services and collaboration between different care providers has increased [3], little is still known about this in relation to the discharge process of older people with dementia from hospital inpatient care [3, 11]. Previous research highlights interprofessional communication across care-providing agencies as a critical issue, showing that limited coordination and inadequate documentation compromise discharges and increase the risk of adverse outcomes [21–23].In Sweden, the responsibility for health and social care is divided between local authorities i.e., regions and municipalities [24–26]—and services are provided by various care providers [26, 27]. Municipalities are responsible for providing social services, and the responsibility for providing health care is shared between regions and municipalities. Regions are primarily responsible for delivering health care, including primary care, acute care, and inpatient care. Municipalities’ responsibilities are limited to municipal health care and rehabilitation provided in the person’s home. Criticism has consistently been raised about the unclear boundaries between these authorities’ responsibilities, which can lead to conflicts about how to proceed with a patient and who holds the primary responsibility [16, 17, 28]. To address this, there is a specific law [26] that requires municipalities and regions to collaborate when a patient is discharged from hospital care. The purpose of this legislation is to promote high-quality health care and social services for individuals who, after discharge, require support from social services or outpatient care. The law specifically aims to ensure that patients are discharged from inpatient care as soon as they are medically ready. Hospital discharges are also briefly mentioned in the Swedish National Guidelines for Care and Services for People with Dementia [29] however, these guidelines primarily refer to the legislation [26] regulating hospital discharge and coordinated care planning.

Hospital discharge processes in Sweden consist of several sequential steps: admission and preparation for the discharge planning meeting, the discharge planning meeting itself, the discharge, and subsequent follow‑up care [30]. According to legislation [26] regulating hospital discharges, the attending physician must assess whether the patient will require continued support after discharge, notify the relevant care providers accordingly, and set a preliminary discharge date. The municipality assumes financial responsibility for the patient after three days. Furthermore, the legislation [26] also clarifies that it is the responsibility of the region and the municipality to develop guidelines and agreements on how collaboration should be organized. This means that the organization of collaboration is decentralized and delegated to local decision-makers (e.g., politicians and local government officials) within the region and the municipality.

In Sweden, there are 21 regions and 290 municipalities. According to the Swedish Constitution, these entities have local self-government, which means that regions and municipalities have significant autonomy in deciding how to carry out their responsibilities and how to allocate their resources [31]. Consequently, there is variation in how different regions and municipalities organize the discharge of patients and what prerequisites exist for collaboration and how coordination of services is organised. As the responsibility is placed upon politicians and local government officials (such as process leaders, strategists, operative managers) as key stakeholders in organizing and providing various services, there is a need to investigate how they perceive collaboration between different agencies and coordination of various services when a person with dementia is discharged from hospital care. To our knowledge, the perspectives of these stakeholders (e.g., politicians and local government officials) have not yet been examined in previous research. Therefore, the aim of this study is to explore local politicians’ and local governmental officials’ perceptions of collaboration and coordination between authorities and their respective care agencies when an older person with dementia is discharged from inpatient care.

## Methods

This study is part of a larger research project that aims to explore collaboration and coordination in Swedish elder care between different authorities and their respective care agencies in relation to hospital discharge for older people with dementia over the age of 65. The project investigates this from the perspectives of multiple stakeholders, including older people with dementia, their relatives, professionals, politicians, and local government officials. The specific focus of the present study is on the perspectives of politicians and local government officials.

In this study, reflexive thematic analysis was utilized, a qualitative approach embedded in a constructivist research tradition [32–34]. The study was designed and conducted in line with the Declaration of Helsinki [35] and is reported following SRQR guidelines ]^36^]. The participating researchers have diverse research backgrounds, including municipal elder care, occupational therapy, citizenship perspectives on dementia, integrated care, and interprofessional collaboration involving older adults, staff, and decision-makers.

### Recruitment

In this study, a maximum variation strategy [37] was utilized to recruit participants to get a diversified data material. To obtain a diverse sample in terms of urban and rural contexts, efforts were made to include participants from larger, and other municipalities located in different regions in Sweden. Regions were selected first, followed by the selection of municipalities within these regions based on urban and rural contexts. Participants were then identified and recruited within these regions and municipalities. Participants holding various strategic-level roles in elder care were sought in order to gain a richer understanding of hospital discharges for older people with dementia. The following criteria were used to include politicians and local government officials: responsible at a strategic level for older people with dementia when discharged from inpatient care with more than one year of experience in the current position.

Before the interview began, written and oral information about the aim and procedure of the study was provided. In total, fifteen people gave their informed consent to participate and were interviewed. Eight people declined to participate or did not respond to our initial contact.

### Data collection

Semi-structured interviews [38] were conducted by members of the research group. Seven of the interviews were conducted in person, and eight via video calls. All interviews were audio-recorded and transcribed verbatim.

The semi-structured interview guide consisted of several questions and covered the following areas: participant demographics; current responsibilities related to the coordination and collaboration of dementia services; how hospital discharge and transfer of responsibility between authorities and care providers were conducted and could be improved; and the extent to which the person with dementia could participate in planning and decisions made in relation to hospital discharge. The questions focused on the participants’ responsibilities at a strategic level as politicians or local government officials.

Data collection was carried out between May and November 2024. An additional interview was conducted in April 2025 to validate the conducted analysis and ensure that no new information emerged that affected the analysis. The duration of each interview ranged from 35 to 79 min, with an average of 55 min.

### Participants

In this study, fifteen participants took part. Nine participants represented seven municipalities, and 6 participants represented four Swedish regions. The municipalities in which the data was collected were located within these four regions. Participants affiliated with municipalities were recruited from three larger municipalities (approximately 100,000 inhabitants), and four other municipalities (approximately 8,000–40,000 inhabitants).

The participants hold various strategic-level roles: local politicians responsible for making decisions on how the municipality or region should be organized to fulfill their responsibilities; strategists who develop proposals or strategies for achieving political goals within established frameworks; operational managers tasked with organizing the agencies operations; and medically responsible nurses or medical responsible for rehabilitation who ensure that municipal medical and rehabilitative interventions are carried out appropriately (Table 1).

**Table 1:** Presentation of the participant’s organizational affiliation

Participants	Municipality	Region
Politicians Strategists Operation managers Responsible for medical care or rehabilitation	2 2 2 3	2 2 2 N/A

### Data analysis

Reflexive thematic analysis [32–34] was used in this study. This analytical framework involves several phases: familiarization with data, data reduction, condensation, coding, and constructing a thematic representation of the phenomenon of interest. The first and last authors read the transcripts and discussed them to gain a comprehensive understanding of their contents. General observations made when going through the data were discussed, and the aim was specified. In the subsequent analysis, relevant sequences to answer the aim were extracted from the data and organized in an Excel^®^ spreadsheet. In total, 480 sequences from the data were extracted.

All sequences were inductively and semantically coded and then sorted into preliminary themes (MA, JÖ). To ensure trustworthiness, these preliminary themes were developed through ongoing analysis and discussions between MA and JÖ who met frequently to ensure a mutual understanding of each other’s analytical interpretations. Furthermore, an additional interview was conducted at this phase. No changes in the thematic representation occurred based on this interview. All themes were then reviewed and discussed by all authors (MA, TT, JÖ) in relation to the entire dataset and the aim of the study. To ensure the trustworthiness of the results, quotations from the interviews are included in the results section to illustrate the analysis and allow readers to assess the analytical claims.

## Results

In the result section, five themes are presented (Table 2) that were pivotal for collaboration and coordination between various agencies when a person with dementia is discharged from inpatient hospital care.

**Table 2:** Overview of themes and key findings

Themes	Key findings
Discharge from inpatient hospital care as a process	Hospital discharge is a complex process requiring collaboration between regional and municipal care providers, beginning at admission and continues after the person has left the hospital.
Agreements Governing Responsibilities in Hospital Discharges	Formal agreements and guidelines between regions and municipalities clarify responsibilities and coordinate tasks when a person with dementia is ready for discharge. These structures can sometimes be challenging to navigate. Strategic forums have played a key role in developing these agreements.
Transferring responsibilities: a physical relocation and an administrative handover	Patient transfer is both a physical relocation and an administrative handover of medical information and responsibilities, emphasizing that discharge should proceed without unnecessary delays to optimize healthcare resources. To ensure safe transitions there is a need for clearer guidelines for sharing patient data and the development of intermediate care solutions.
Strategic workforce planning for sustained staff competence	Strategic workforce planning is crucial for ensuring staff to have necessary competencies to coordinate and collaborate effectively across healthcare and social services, particularly in dementia care. Identified challenges was high staff turnover, inconsistent practices, and a lack of specialized skills in dementia care.
Ensuring the individual’s representation in the discharge process	A person-centred approach is essential for ensuring that individuals with dementia are represented during discharge from inpatient care. Strategic support was highlighted to ensure that older people with dementia receives appropriate care who might decline services or are unable to advocate for themselves receives appropriate care.

### Discharge from inpatient hospital care as a process

All participants described the discharge from inpatient hospital care as a process involving several professionals from different authorities and care providers, requiring collaboration among staff and coordination of services over an extended period. Participants with a regional perspective explained that the process begins already at the point of admission, where initial planning and early contact with relevant care providers are essential. Participants with a municipal perspective emphasized that the process does not end when the person leaves the hospital. Instead, it continues, as it requires, for example, ongoing staff training, follow-up on interventions, and sustained involvement from both regional and municipal outpatient services. Regional participants reported that their organization had previously faced challenges in identifying the appropriate contact person within the municipality at the point of discharge. This lack of clarity sometimes complicates coordination and delays necessary follow-up care. However, efforts have in the last decade been made to address this issue, including the introduction of designated discharge coordinators and the establishment of written handover procedures to ensure clarity and continuity throughout the discharge process.

“*Ideally*,* the transition of responsibility between care providers should be so seamless that the individual does not even notice it has occurred. The person should receive the support they need regardless of who is formally responsible at any given time /…/ Whether or not this vision will ever be fully realised*,* I cannot say. But I believe this is how the system ought to function—especially for this patient group.”* (Participant 1, official, other municipality).

Given that discharge from hospital is complex, participants emphasized the importance of carrying it out consistently for all patients. This helps reduce the risk of the process being driven by particularly committed individuals acting as care coordinators, or patients being discharged prematurely. The participants described ongoing efforts to refine discharge procedures to establish a system perceived as safe and reliable for all older people. Achieving this requires timely involvement from the appropriate care providing agency, supported by well-established relationships that help anticipate and prevent potential challenges. However, participants also recognized that the discharge process presents considerable challenges due to the cognitive impairments associated with dementia. Some politicians and operational managers acknowledged the need to adapt the process to better accommodate these patients. In contrast, strategists often opposed such adaptations in the interviews, arguing that tailoring the process to specific diagnoses would be too complex, as different target groups would require different types of adjustments.

*“If we were to start looking at creating a different variant of the process*,* then I think we would need to make many variations*,* given that there is other—how should I put it—other diagnoses and so on that might also need to be considered a bit differently. Therefore*,* if you ask me*,* I believe that we need to have a unified process as a foundation*,* but then perhaps the steps need to be considered in slightly different ways. However*,* having the same overall process is what I would say is preferable”.* (Participant 2, official, large municipality)

Participants with extensive experience noted that the length of inpatient care has gradually decreased over time. According to the participants, care planning at discharge is now based on what they perceive as significantly shorter hospital stays. They generally agreed that it is appropriate for older people with dementia to remain in the hospital for as short a time as possible, to reduce the risk of additional infections and to free up hospital beds. However, they also pointed out that this shift means that hospital staff have less time to gather information and conduct thorough assessments before discharge, which may compromise the quality of discharge planning and the continuity of care.

*“I believe it is fundamentally about ensuring that individual needs are properly addressed. /…/ It is crucial that staff have sufficient time to carry out the necessary steps—such as assessing cognitive function and identifying care needs—so that a fair and accurate baseline is established for the individual/…/ I do not believe that the current system is sufficiently equipped to ensure a smooth and effective process”* (Participant 3, official, region).

### Agreements governing responsibilities in hospital discharges

According to the participants, when a person with dementia is ready for discharge, a range of agreements guide the responsibilities of different authorities and the coordination of tasks. To ensure clarity and consistency, participants emphasized that routines should be based on formal agreements and guidelines, both within and between regions and municipalities. While these structures are generally helpful, they can occasionally present challenges.

*“We’ve observed that when people find the process complicated*,* they tend to come up with alternatives that don’t align with the established procedures”* (Participant 2, official, large municipality).

According to the participants, the development of agreements has been ongoing for several years, often relying on personal contacts and shared strategic-level forums. Almost all participants—except operational— managers took part in these forums. However, those responsible for medical and rehabilitation services expressed uncertainty about their mandate within these strategic discussions. Instead, they tended to contribute with an operational perspective from a municipal point of view and relayed information back to their organizations as needed.

The participants highlighted the complexity that arises when a region wishes to change or introduce a new procedure, as it must be established in collaboration with several municipalities within that region. Some participants described that all municipalities within their region had come together as one, both to streamline coordination within the region and to strengthen their collective voice in negotiations. On the other hand, if a municipality wish to introduce new routines, then there is no single representative within the regional authority who can speak on behalf of for example all primary care centres, as several of them are privately operated. Therefore, the municipality needs secure approval from all primary care centres within the municipality individually.

*“The region distances itself from this responsibility*,* stating that it is not their role to speak on behalf of the private providers. While it is understandable that they may not be able to represent them directly*,* they could at least coordinate efforts across all affiliated primary care centres /…/ Then they could present a unified position to us*,* saying*,* ‘This is our shared view*,*’ and we*,* in turn*,* could respond with a unified voice. Naturally*,* this would involve compromise. However*,* at present*,* no one holds the mandate to engage in that kind of dialogue with us.”* (Participant 4, politician, large municipality).

The politically adopted agreements between regions and municipalities—concerning, for example, the three-day discharge rule—is largely based on existing legislation governing safe discharge from inpatient care. According to the participants, many elements of an agreement do not differ significantly from what is already stipulated by the Act on Collaboration in the Discharge Process from Inpatient Health Care (Swedish Code of Statutes 2017:612) to ensure patient safety during the discharge process. As previously mentioned, legislation stipulates that municipalities have three days to arrange for a patient’s discharge from the hospital. Participants noted that municipalities can argue that a patient is not ready for discharge under current conditions and may request a more comprehensive assessment or additional support measures before proceeding. Despite this possibility, it appears that municipalities are not fully utilizing this option to delay discharges when necessary.

*“There was a regulatory change a few years ago. I believe it was a national change in the legislation—now you have three days to discharge a person from the hospital*,* which wasn’t a limitation before. Also*,* I think the municipality isn’t always making full use of the flexibility that exists. In several cases*,* we could actually say: ‘No*,* this person cannot be discharged under these conditions. We need to carry out a more thorough assessment first*,*’ or ‘There’s a need for training or other preparatory measures.’ So that’s an option that municipalities might be underutilizing/…/ I could imagine that’s the case*,* especially when it comes to someone with severe cognitive impairment.”* (Participant 1, official, other municipality).

### Transferring responsibilities: a physical relocation and an administrative handover

The participants expressed that transferring a patient involves both a physical relocation, and an administrative handover of medical information and responsibilities. When a patient is assessed as medically ready for discharge, the process should proceed without unnecessary delays. By referring patients to appropriate care settings, instead of keeping them in hospitals, the participants stressed that healthcare resources can be used more efficiently. In hospital settings, there is a policy-driven directive to initiate discharge and ensure hospital bed availability. According to participants, this is also in the interest of municipalities, as delays in the discharge process prevent hospitals from admitting new patients. Participants highlighted that this issue becomes particularly pronounced before weekends, when hospital capacity needs to be freed up, leading to frequent discharges on Fridays. In relation to this, participants expressed concerns that when discharges are rushed, critical aspects—such as the availability of staff, medications, or assistive devices—may be overlooked. Furthermore, crucial information may not be adequately communicated to caregiving staff.

*“You might receive a call at 1 p.m. on a Friday*,* informing you that double staffing will be required five times a day throughout the entire weekend. That means chasing staff*,* ensuring that the necessary interventions are in place – it’s simply part of my everyday work.”* (Participant 5, official, large municipality).

During the summer months and major holidays, hospital bed availability is also reduced, placing additional strain on municipal healthcare services. Participants stressed the importance of effective collaboration between regional authorities and municipalities during these periods, with an emphasis on innovative care models that ensure continuity of care. Both hospitals and municipalities face organizational challenges that often lead to a shifting of responsibilities rather than collaborative problem-solving. To address these challenges, participants emphasized the need for greater transparency and flexibility in the collaboration between regions and municipalities.

*”There are always lead times associated with the resources you need—whether that be specific competencies*,* delegation processes*,* or ensuring staff are adequately trained. For example*,* within the nursing field*,* a patient may be discharged with advanced medical equipment such as a tracheostomy or stoma care devices. In such cases*,* staff must be trained and introduced to the equipment and procedures to ensure they can manage the situation effectively. This is essential to avoid the need for a nurse to visit multiple times per day to carry out the treatment. In order to make home-based care feasible*,* certain tasks often need to be delegated*,* and that process takes time”* (Participant 6, official, large municipality).

According to the participants, being medically treated is not synonymous with being ready for discharge—ensuring a safe transition requires that all surrounding factors are appropriately addressed. Participants with a regional perspective also emphasized the importance of adapting to the varying conditions of different municipalities to ensure the best possible outcomes for patients. Both regional and municipal representatives noted the need for an intermediate care setting between hospital care and the home or nursing home environment—commonly referred to as “short-term care.”

*”The discussion often centres around the question of who is considered medically fit for discharge*,* and that threshold has shifted over time. As the population has aged and the number of older individuals has increased*,* the pressure on the system has grown accordingly. This raises the question: has there been a tendency in the past for the region to take on a greater share of responsibility*,* meaning that patients were kept in hospital longer than strictly necessary? If so*,* municipalities may not have previously needed to assume as much responsibility for post-discharge care. Alternatively*,* is it now the case that the region is discharging patients who are too unwell*,* placing an inappropriate burden on municipal services? I believe the reality lies somewhere in between—it’s a matter of give and take on both sides”* (Participant 7, politicians, region).

Information transfer is another crucial factor regarding collaboration raised by the participants. Upon discharge, patient needs may change, rendering previous hospital-provided information outdated. The region and the municipalities do not have the same Electronic Medical Record, meaning that it is not possible to read each other’s documentation.

*” One of the ongoing challenges is that we operate with different electronic health record systems*,* and as things currently stand*,* that is unlikely to change. This can make it somewhat difficult when it comes to referrals and responses to referrals.”* (Participant 8, official, large municipality).

Almost all participants described, from a strategic perspective, that it is possible to send messages and share patient information through an Electronic Health Record (EHR) system. However, this requires established guidelines not only for documenting which data should be included, but also for how documentation can be shared between different authorities and care providers. The participants emphasized that the development of such guidelines must comply with relevant legal frameworks and be governed by access control. Furthermore, they highlighted the need for legislative changes to enable this.

*”The process is extremely cumbersome*,* and I genuinely believe this is an area where policymakers must take action—and we are*,* but it requires sustained effort throughout the system. At present*,* we cannot access each other’s electronic health record systems. We are unable to share information directly.”* (Participant 7, politician, region).

### Strategic workforce planning for sustained staff competence

Strategic workforce planning was emphasized as essential to ensure that staff have the necessary competencies to effectively coordinate and collaborate within healthcare and social services. Participants described this as a complex and multifaceted challenge. Contributing factors included high staff turnover, the ongoing need to train and integrate new employees, difficulties in maintaining consistent work practices, a lack of staff with appropriate educational qualifications, and the low attractiveness of careers in dementia care. A particularly emphasized area is the need to plan for and address the needs of individuals with Behavioural and Psychological Symptoms of Dementia (BPSD), as well as younger individuals with dementia. In the case of the latter, the involvement of family members often adds complexity to the care situation, thereby increasing the demand for staff with specialized skills.

In addition to competence, participants highlight the need for appropriate types of resources for older people with dementia. Ensuring that these resources are sufficient requires collaborative, team-based approaches involving both municipalities and regional authorities. However, this is also a matter that demands attention within each individual municipality and region. For rural municipalities, this may pose a particular challenge, as described by one participant:

*” We are*,* after all*,* located in a rural area*,* and that presents challenges [in relation to workforce planning] in meeting the demands placed upon us. It feels like an uphill struggle*,* quite frankly. I believe what is needed includes targeted educational initiatives*,* but also*,* crucially*,* stronger national incentives to support and strengthen this professional group [municipal health and care staff]”* (Participant 9, politician, other municipality).

A central aspect of strategic workforce planning, according to the participants, is to create conditions for collaboration between different professions and care providers. For example, the need for physicians with experience and competence in geriatrics and cognitive impairment was particularly emphasized. Participants with a municipal perspective also stressed that physicians should have knowledge of care home environments and home-based care, in order to foster a sense of security for individuals living with dementia in their own homes. Furthermore, several participants pointed out that municipal managers without healthcare backgrounds may lack sufficient insight into medical needs. This highlights the importance of delegating healthcare-related decision-making to qualified professionals, while maintaining managerial oversight at the organizational level.

Another way to ensure strategic workforce planning, according to the participants, is to establish structures that support interdisciplinary collaboration. One initiative described is the establishment of Dementia Teams, composed of professionals from various disciplines. These teams engage with individuals and their families at an early stage and provide support throughout the different phases of the illness—for example, during hospital discharge and in the planning of home-based care interventions. Dedicated dementia teams and specialists, such as specialised dementia care nurses, can offer expert support, particularly during transitions from hospital to home. Dementia centres also serve as valuable resources in addressing challenges related to inter-organizational coordination.

### Ensuring the individual’s representation in the discharge process

A person-cantered approach was described as crucial for coordinating services when a person with dementia is discharged from inpatient care, ensuring that the person is represented in the process. Traditional hospital care often focuses exclusively on immediate medical concerns; however, participants stressed that there is a growing necessity to integrate a patient’s broader lived experience into care strategies. Such a paradigm shift requires time and structured implementation. Targeted government grants could play a pivotal role by fostering collaborative efforts and ensuring measurable progress.

*“There is considerable work to be done*,* particularly in terms of leadership within both integrated and person-centred care. I would especially highlight the need for development among managers of care units (SSA act)*,* that is*,* the service providers. These managers often lack experience in this area. Another challenge lies in the perception among registered professionals*,* who frequently believe they are already practicing person-centred care*,* when in fact their approach tends to be more profession centred. Their focus is often on what they can offer*,* rather than on what the individual truly needs. I would argue that registered professionals can be the most difficult to engage*,* precisely because they believe they are already aligned with the principles of person-centred care”* (Participant 1, official, other municipality).

A trusting and supportive relationship between staff and the care recipient is essential to ensure that someone takes responsibility for identifying and addressing needs that may be overlooked during discharge planning. In relation to this, participants stressed that it is necessary to have familiar and competent staff close to the users, who know them personally, both in terms of addressing their needs and handling these needs according to their preferences. However, this might put staff in a precarious position where doing what is best for the person might be considered wrong by the system.

*“And I think that we have fantastic staff close to the users who solve these issues*,* even though it essentially means that they are doing something wrong. […] We put staff in a position where doing what is best and the only reasonable thing is wrong”* (Participant 9, politician, other municipality).

It was commonly described by the participants that individuals with dementia often lack the capacity to represent their own interests during discharge planning, leading to decisions being made by others. The heterogeneity of older people with dementia makes it challenging to determine when someone else should assume responsibility for making decisions on their behalf.

*“That’s why I think there must be these kinds of trustworthy structures because we want the best for these people. And it’s not always certain that they themselves understand what’s best for them”* (Participant 7, politician, region).

From a strategic perspective, participants highlighted this as a vulnerable population of older people who need support from health care and social services. Still, this population often declines services, resulting in them not receiving the necessary assistance or refusing to allow staff to administer services. Participants also noted the risk of authorities exploiting persons who declined services by hiding behind the principle of self-determination, which can leave these individuals without adequate support.

*I think we need to ensure that legislation removes the possibility for municipalities or regions to say*,* “Well*,* Pelle or Agda didn’t want our help*,* so we stepped aside and let this tragic event happen.” Formally and legally*,* they have actually done the right thing by respecting the individual’s will.* (Participant 9, politician, other municipality)

These examples illustrate the ethical and practical dilemmas in collaboration and coordination during the discharge process when striving to ensure person-centred care for older people with dementia. To address these challenges, participants emphasized the need for trustworthy structures and legislative clarity that support professional responsibility without compromising the individual’s rights or well-being.

## Discussion

In this study, we engaged strategic-level representatives from both municipalities and regions in Sweden to explore their perceptions of collaboration and coordination between authorities and care agencies when a person with dementia is discharged from hospital care. This stakeholder group holds a central role in Sweden, with elected politicians responsible for strategic decision‑making, budgeting, and prioritization, and civil servants responsible for operational management, implementation, and the development of agreements across different authorities and care providers. Despite their pivotal position in shaping discharge practices, this group has not previously been the focus of research concerning hospital discharges for older people with dementia [3]. By focusing on this under-researched yet strategically important stakeholder group, this study contributes new and valuable insights into the discharge process for older people with dementia.

The findings, in line with previous research [30], portray hospital discharge as a continuous process unfolding over an extended period of time and shaped by multiple factors, including legislative frameworks, service availability, and patients’ preferences. The study further demonstrates that actors in strategic positions also conceptualise discharge as a process; however, their perspectives vary depending on both their role and the authority with which they are affiliated. For example, the results indicate that politicians call for a dedicated discharge process for people with dementia, whereas strategists emphasise the importance of applying the same process regardless of diagnosis. Given that dementia affects individuals’ cognitive abilities [7] and their capacity to participate in decisions regarding health and social care services [39, 40], it may be appropriate to either develop a specific discharge process or redesign existing ones to ensure that they genuinely accommodate the needs of all patient groups. Discharge was conceptualised as an ongoing process, but differences in how this process was described reflected organisational affiliation. Participants with a regional perspective tended to define the beginning of the process as occurring at hospital admission, whereas those representing a municipal perspective emphasised what happens when the individual leaves the hospital. This divergence is not unexpected, given the division of responsibilities between these authorities [24–26]. However, to advance collaboration and coordination of services, there remains a need to further develop mutual understanding of each other’s responsibilities, as well as the conditions and constraints under which integrated care for patients can be provided.

Older people with dementia are commonly described as a vulnerable group with complex health and care needs—and, to some extent, dependent on the goodwill of others to receive adequate support [39, 41, 42]. Adding to this complexity is the fact that responsibility for meeting complex health and care needs often falls to multiple care agencies operating under different legislative frameworks. This fragmentation of health and care services [14, 15, 43] may make it difficult for older people with dementia to navigate the system [3], thereby increasing their dependence on others — both professionals and family members. Our findings further suggest that the perspective of the person with dementia may be overlooked when professionals become too rigid in their interpretations of legislation—or even use it as a shield—particularly in situations where the individual declines offer of support. When politicians and local government officials develop agreements governing responsibilities in hospital discharges, this must be considered. Therefore, there is a need to reinforce mutual accountability and responsibilities amongst professional and organizational boundaries [44].

The fragmentation of health and care services has previously been discussed in the literature [14, 15, 43]. Our findings build on this work by illustrating the complexity of inter-authority arrangements, particularly with regard to agreements governing how services are organized. For example, all but one Swedish region (i.e., Gotland) include several municipalities, and a single municipality may encompass multiple primary care centres operated by different healthcare providers under regional responsibility. Consequently, all parties involved in the discharge process need to establish agreements that clearly define procedures and responsibilities related to hospital discharge. As highlighted in previous research [45], this underscores the need for flexibility among and between the stakeholders involved. Furthermore, regions may need to develop context-specific procedures for how hospital discharge is conducted, depending on the patient’s municipality of residence. This adds to earlier research by drawing attention to how local self-government in Sweden shapes hospital discharge processes for older people with dementia. The findings of this study show that politicians and local government officials express concerns about inpatient care, as it often involves short hospital stays and rapid discharge planning. These circumstances place demands on the development of new working methods that ensure the needs and wishes of older people with dementia are acknowledged and guided to the continued planning of health and care services. This study adds the need for a representative who knows the person’s wishes and past experiences (e.g., life history, medical situation), to ensure these are considered in decision-making and that older people with dementia voices are heard. Previous studies have highlighted care coordinators as key stakeholders, that often focus on either health or social care needs depending on their organizational affiliation or professional background [46–48].

Furthermore, based on our findings, there is a shared need among both regions and municipalities to collaborate and coordinate services in order to ensure the efficient use of available resources. This need includes ensuring the availability of hospital beds for newly admitted patients, which in turn requires the discharge of those already admitted. According to the findings, politicians and local government officials expressed concern that individuals are sometimes discharged despite not being medically ready. This is particularly critical given that previous research has shown that people with dementia have higher rates of hospital readmission and an increased risk of mortality, pointing to potential gaps in post-discharge care [49]. These findings underscore the need for collaboration to develop jointly designed alternatives that prevent individuals who are not medically ready from being discharged prematurely, remaining in hospital longer than necessary, or falling between services when they do not qualify for existing options such as short-term residential care. There is therefore a need for solutions beyond what currently exists for older people with dementia. Several participants emphasized that a potential solution could be the establishment of new accommodation, jointly staffed by personnel from both the region and the municipality. This adds to existing arguments that call for allocating resources at policy-level to foster interprofessional collaboration [45].

Politicians and local government officials participating in this study raised concerns about a high turnover of staff making it difficult to establish a consistent organizational culture around how hospital discharge is carried out in practice—both within regional and municipal health and social care. Furthermore, trained staff are needed—professionals who are genuinely interested in working with older people with dementia using a person-centred approach during hospital discharges.

### Methodological consideration

A maximum variation strategy [31] was employed to include participants holding various strategic-level roles, to gain a richer understanding of the process of discharging older people with dementia from inpatient care. As this is an exploratory study, efforts were made to include representation from different levels of strategic decision-making. Consequently, the participants’ roles ranged from political leadership to operational managers responsible for implementing decisions within the local administration. Furthermore, participants were selected from several regions municipalities to ensure a diverse dataset that is not tied to a specific local context or organizational model for hospital discharge. Therefore, the findings of this study are not intended to be transferable or generalizable. Rather, the aim is to explore local politicians’ and local governmental officials’ perceptions of collaboration and coordination between authorities and their respective care agencies when a person with dementia is discharged from inpatient care.

Several participants shared previous work experience, as well as personal experiences involving family members with dementia who had been discharged from inpatient care. These statements were not included in the analysis, as they were not expressed in relation to the participants’ roles as strategic-level decision-makers and thus fell outside the scope of this study. Nonetheless, it is possible that their personal experiences influenced their general perceptions, as it can be difficult to fully separate one’s personal self from one’s professional or political role.

Previous reviews on decision-making for older people with dementia—within both clinical and social care services—have shown that research in this field often lacks a clear presentation of contextual factors [39, 50]. As there are various health and social care settings with various policies and practices in different countries, the context must be presented and incorporated in the analytical work conducted [50] to ensure trustworthiness. Therefore, the legislative context of the study is thoroughly outlined in the method section.

## Conclusion

This study highlights the intricate challenges of coordinating care for individuals with dementia during hospital discharge, as perceived by strategic-level stakeholders in Sweden. The complexity of inter-authority structures—where regions encompass multiple municipalities and care providers—creates variability in procedures and responsibilities, often leading to uncertainty and fragmented transitions. Our findings underscore the need for improved mutual accountability across care providers and authorities. Strengthening collaboration and consistency across authorities is essential to ensure equitable, person-centered care for older people with complex health and social care needs.

## Data Availability

This article includes illustrative quotations drawn from the data generated and analyzed during the study. The data supporting the findings of this study are available from the research lead, JÖ, upon reasonable request.
